# Fused sphere carbon monoliths with honeycomb-like porosity from cellulose nanofibers for oil and water separation†

**DOI:** 10.1039/d0ra08950h

**Published:** 2021-01-08

**Authors:** Mark Adam Ferry, Jun Maruyama, Taka-Aki Asoh, Hiroshi Uyama

**Affiliations:** Osaka University Graduate School of Engineering, Division of Applied Chemistry 2-1 Yamadaoka Suita, Osaka 565-0871 Japan uyama@chem.eng.osaka-u.ac.jp; Osaka Research Institute of Industrial Science and Technology, Research Division of Environmental Technology 1-6-50 Morinomiya Osaka 536-8553 Japan maruyama@omtri.or.jp

## Abstract

Carbon monoliths with a unique hierarchical surface structure from carbonized cellulose nanofibers were synthesized in pursuit of developing carbon materials from sustainable natural resources. Through a 2-step hydrothermal – carbonization method, TEMPO-oxidized cellulose nanofibers were turned into carbon-rich hydrochar embedded with polystyrene latex as template for 80 nm-sized pores in a honeycomb pattern, while the triblock copolymer Pluronic F-127 was used for a dual purpose not reported before: (1) an interface between the cellulose nanofibers and polystyrene particles, as well as (2) act as a secondary template as ∼1 μm micelles that form hollow carbon spheres. The use of nanofibers allowed more contact between the carbon spheres to coalesce into a working monolith while optimizing the pore structure. Oil–water separation studies have shown that carbon monoliths have high adsorption capacity due to surface area and hydrophobicity. Testing against commercially available activated carbon pellets show greater performance due to highly-developed macropores.

## Abstract 2

Carbon monoliths with a unique hierarchical surface structure from carbonized cellulose nanofibers were synthesized in pursuit of developing carbon materials from sustainable natural resources.

## Graphical abstract

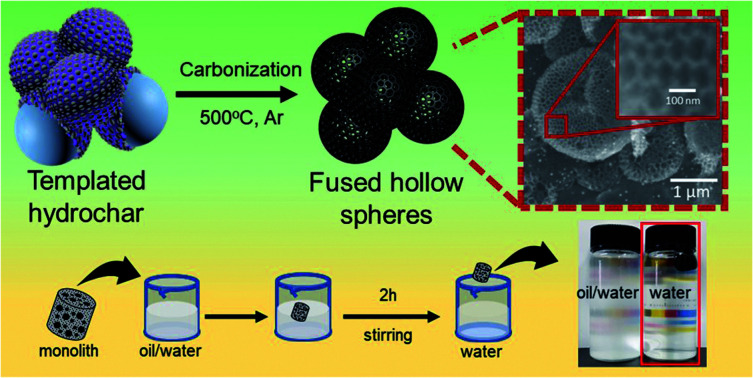

## Introduction

Porous carbon materials are known to be optimal for adsorption,^1^ electrochemical,^2^ and catalyst media^3^ applications due to its inertness, surface morphology and high surface area. Precisely due to the stability of carbon, developments in improving the performance of carbon-based products have been usually on increasing the surface area or determining methods of controlling the microstructure^4,5^ instead. Achieving high porosity in carbon materials is normally performed using a 2-step activation process using KOH^6^ or H_2_O steam^7^ as the oxygen source to induce micropores on the surface of the carbon. While high surface area can be achieved, longer processing time and processing requirements such as powderizing the sample for sufficient contact with the oxidant limits processability and the types of products possible with this type of carbon material. Another approach is by using templates such as silica particles,^8–11^ but the process is still lengthened by the template removal step. In contrast, using templates that degrade during carbonization reduce the steps required to induce porosity, and also allow the bulk material to retain its form prior to carbonization.

Carbon sources vary, but one particular current interest is the use of biomass and natural polymers. Sources from biomass such as discarded apricot shells^12^ and treated food waste^13,14^ have been reported. Variability in the composition of biomass, however make investigating carbon synthesis more difficult, which is why carbon from more homogeneous biomass sources are preferred. Cellulose-based materials are a promising carbon source for its abundance, low cost, and more predictable composition. Most cellulosic biomass is composed of cellulose, hemicellulose, and lignin, of which the behavior of cellulose and hemicellulose during carbonization is well-known.^15^ Commercially available cellulose has high crystallinity, resulting to compact packing of the fibers. Furthermore, H-bonding between polymer chains prevents facile uniform dispersion and effective templating. In order to separate the individual fibers, oxidation is performed on the hydroxy groups of the glucose residue to induce static repulsion, forming nanofibers.^16,17^ Selective oxidation of the C6 carbon to form a carboxy group by using (2,2,6,6-tetramethylpiperidin-1-yl)oxyl or TEMPO has been devised by Isogai *et al.* which result to fiber diameter of as small as 5 nm.^16,18^

Inducing a templating effect on carbon materials, whether it is through silica or polymer particles, requires a pre-treatment step wherein the template is mixed and adhered to the carbon source before carbonization. Hydrothermal treatment (HTT) is a common treatment step for biomass conversion that degrades and homogenizes the precursor material under high temperature and pressure in order to form hydrochar. HTT results in homogeneous materials which could trap any dispersed material in the hydrothermal vessel that did not decompose under HTT conditions. In a study by Kubo *et al.*,^4^ polystyrene particles dispersed in a d-fructose solution with Pluronic F-127 (PF 127) showed that polystyrene nanoparticles stay inert under HTT and can be dispersed in the hydrochar. Because hydrochars are carbon-rich, carbonizing hydrochar becomes more convenient by removing much of the usual decomposition products and water.

The degradation pathways of cellulose and other polysaccharides during hydrothermal treatment have already been well-studied, and similarities in the polymer – glucose residues – have resulted in a similar mechanism^19,20^ and kinetics.^21^ Chain scission into monosaccharides or disaccharides first occurs, followed by ring opening and subsequent oxidation^20^ at random points in the glycoside ring. From this point the pathways diverge according to the phase where it occurs: the aqueous phase and the organic phase. In the aqueous phase, furfural and 5-hydroxymethylfurfural (5-HMF) are formed from the oxidation of glucose and fructose. 5-HMF and furfural, which are dissolved in the organic phase, further oxidize into phenols. These aromatic species then crosslink and eventually precipitate out of the liquid phases to form the hydrochar bulk. The other smaller degradation products either volatilize, remain in the solvent, or precipitate into the hydrochar. It is notable however that not all polysaccharides form a hydrochar monolith. Depending on the HTT conditions and the precursor material, precipitates or even microspheres^19,22,23^ are formed. It is established that polysaccharides under hydrothermal reaction form hydrochar from aromatic compounds formed *in situ* from glucose residue degradation products.^20^

Utilizing HTT as the pre-treatment step for cellulose-based activated carbon while incorporating removable templates gives rise to controllable porosity and macrostructure. Uses such as desalination,^1^ CO_2_ capture,^24^ pollutant sequestration,^25^ and drug delivery^26^ are reported, but one application where multimodal carbon could be effective is the adsorption of adsorbates ranging from metals,^27^ dyes,^28^ organic compounds,^29^ and hydrocarbons^30,31^ for environmental clean-up. High porosity of carbon allows for high adsorption capacity, thus making it an effective material for oil–water separation.^32,33^

In this work, porous carbon was synthesized using the hydrothermal-carbonization method, with polystyrene particles dispersed in a cellulose nanofiber mixture as the matrix. Characterization of the carbon monolith (CM) were performed to determine the optimal ratio of TEMPO-oxidized cellulose nanofibers (TOCN), polystyrene latex (PSL), and Pluronic F-127 (PF 127), as well as the carbonization temperature of the hydrochar on the pore structure. Oil adsorption capacity (*q*_e_) was evaluated, and compared with commercially available activated carbon pellets.

## Experimental section

### Materials

The list of reagents used are as follows: cellulose powder (C_6_H_12_O_5_)_*n*_ from Nacalai Tesque, NaBr from Sigma-Aldrich, (2,2,6,6-tetramethylpiperidin-1-yl)oxyl or TEMPO (C_9_H_18_NO) from Tokyo Chemical Industry, 8.5–13.5% NaClO from Nacalai Tesque, sodium dodecyl sulfate or SDS (NaC_12_H_25_SO_4_) from Wako Pure Chemical Corporation, 0.5 M sodium hydroxide solution (NaOH) from Sigma-Aldrich, styrene monomer (C_8_H_8_) from Nacalai Tesque distilled once under low vacuum, ammonium persulfate or APS ((NH_4_)_2_S_2_O_8_) from Sigma-Aldrich, Pluronic F-127 from Sigma-Aldrich, activated carbon pellets from Wako Pure Chemical Corporation, tetrachloroethylene (C_2_Cl_4_) from Tokyo Chemical Industry, octanoic acid (C_7_H_15_COOH) from Nacalai Tesque, and isooctane (C_8_H_18_) from Nacalai Tesque. Except for styrene, all other reagents were reagent grade, and used as received.

### Characterization

Field Emission Scanning Electron Microscopy (FESEM) micrographs were taken from a JEOL JSM-6700F microscope. Scanning Electron Microscopy (SEM) micrographs were obtained using a Hitachi SU3500 microscope. Infrared Spectroscopy (IR) spectra were taken by a Nicolet iS5 Attenuated Total Reflectance spectrometer. IR spectra taken using the KBr pellet method used a Jasco FT/IR-4100 infrared spectrometer. Thermogravimetric Analysis (TGA) was measured using a SII TG/DTA7200 instrument. Dynamic Light Scattering (DLS) spectra were taken by an Otsuka ELSZ-2000 Particle size Analyzer. Brunauer–Emmett–Teller Surface Area Analysis (BET) was taken from a Quantachrome NOVA 4200e Surface Area and Pore Size Analyzer. All samples were dried in vacuum at 70 °C for 24 h before BET analysis. X-ray photoelectron spectroscopy (XPS) spectra were taken by a JEOL JPS-9010MC XPS instrument with monochromatic AlKα-radiation. CasaXPS Version 2.3.15 was used for analyzing the C 1s narrow XPS profile. The water contact angle was controlled to drop 1 μm water by DropMaster DM 300. FAMAS was used to analyze the water contact angle images.

### Synthesis and characterization of TEMPO-oxidized cellulose nanofibers (TOCN)

Cellulose (4.0 g), NaBr (0.768 g), and TEMPO (0.075 g) were added to water (300 mL) and stirred for 1 h until homogeneous. NaClO (18 mL of 8.5–13.5% solution) was then added dropwise. The mixture was stirred overnight while maintaining the pH at 10.5 using an autotitrator with 0.5 M NaOH to maintain excess ^−^OH as oxygen source for oxidation. When the reaction is complete indicated by the color change from yellow to white, the cellulose pulp was separated from the liquid *via* centrifugation at 4000 rpm. The white pulp re-dispersed in 40 mL water, then centrifuged again at 4000 rpm. The process of redispersion and centrifugation was repeated 4 times to clean the pulp. The purified cellulose was then diluted with water to around 1% wt/wt, then fibrillated using a blender for 0.5 h. An average size of 53 ± 15 nm was calculated from SEM images of TOCN (Fig. S1†).

### Synthesis and characterization of polystyrene latex (PSL)

SDS (1.5 g) was dissolved in water (258 mL), then styrene (30 mL) was added dropwise. APS (0.01 g) dissolved in water (12 mL) was added and stirred, then the reaction flask was purged with N_2_ (g) for 0.5 h. The reaction flask was then placed in a 70 °C water bath and stirred for 3 h. The PSL was placed in a 50 kDa dialysis tube, and dialyzed for 5 days with regular water changes until no trace of styrene monomer was detected. The PSL diameter was found to be at 66 ± 55 nm using DLS (Fig. S2†).

### Synthesis of the carbon monolith (CM)

TOCN dispersion (10 g of the 1% wt/wt, corresponding to 0.1 g dry TOCN), PSL, Pluronic F-127 (PF 127), and water for a total volume of 12.8 mL was mixed for 1 h until homogeneous. The viscous mixture was then transferred to a hydrothermal reaction vessel and placed in an oven for 48 h. The flask was cooled to room temperature, then the resulting solid called the hydrochar monolith (HM) was separated from the supernatant. HM was washed with EtOH 3 times and then with water for another 3 times. The hydrochar was then freeze-dried for 48 h. The dried hydrochar was placed in a furnace under Ar_(g)_ atmosphere with a constant flow rate of 75 mL min^−1^. The temperature was raised with a heating rate of 5°C min^−1^ to a temperature *T*_hold_, maintained for 2 h, then allowed to cool down overnight. The final product was a carbon monolith (CM). The list of experiments performed and their corresponding conditions are listed in Table 1.

**Table 1:** List of samples. Samples after HTT are labelled as HM-XXX, and samples after carbonization are labelled CM-XXX, where XXX is the code listed below

Code	PSL/g	PF 127/g	HTT temp/°C	*T*_hold_/°C
**Variation of PSL content**				
S1	0.016	0.010	180	500
S2	0.032	0.010	180	500
S5	0.080	0.010	180	500
S10	0.160	0.010	180	500

**Variation of PF 127 content**				
F1 (=S5)	0.080	0.010	180	500
F2	0.080	0.020	180	500
F5 (=H180, C500)	0.080	0.050	180	500
F10	0.080	0.100	180	500

**Removal of polymer components for determining structure formation**				
SxF1	—	0.010	180	500
SxF5	—	0.050	180	500
S5Fx	0.08	—	180	500

**Variation of HTT temperature**				
H180 (=F5, C500)	0.080	0.050	180	500
H200	0.080	0.050	200	500

**Variation of carbonization temperature (*T***_**hold**_ **)**				
500c (=F5, H180)	0.080	0.050	180	500
600c	0.080	0.050	180	600
700c	0.080	0.050	180	700
800c	0.080	0.050	180	800
900c	0.080	0.050	180	900

### ImageJ analysis of SEM and FESEM images

The pixel-distance ratio is set from the known scale bar. The image is then binarized to black as the target particle or pore, and white as the background. The Analyze Particle function was used, with an estimated particle size and circularity input to prevent the inclusion of erroneous particles in the sample population. A sample population is kept to be at least 30 per analysis. The diameter is then calculated assuming that the pore is circular.

### Adsorption experiments

The adsorption adsorbate, calibration (Fig. S3†), extraction, and analysis were based on the oil and grease determination method through mid-infrared method.^34^ All adsorption experiments were conducted in 50 mL silicone-sealed brown borosilicate glass to prevent oxidation and contamination. 50 mL of 5 mg mL^−1^ mixture of 1 : 1 octanoic acid and isooctane dispersed in water was stirred at 150 rpm for 0.5 h then a CM of 0.02 g was placed inside. The CM was removed after 120 min, then the remaining oil was extracted using C_2_Cl_4_. The extract was diluted then analyzed using a Jasco FT/IR-4100 spectrometer at 2933.2 cm^−1^. The FTIR conditions were set at a of resolution 8 cm^−1^, 64 scans, quartz cell with path length of 10 mm (Fig. S4†). Comparative studies of the synthesized monoliths with commercially available activated carbon pellets were also performed using the same conditions.

## Results and discussion

### Effect of variation of PSL content

**Fig. 1:**
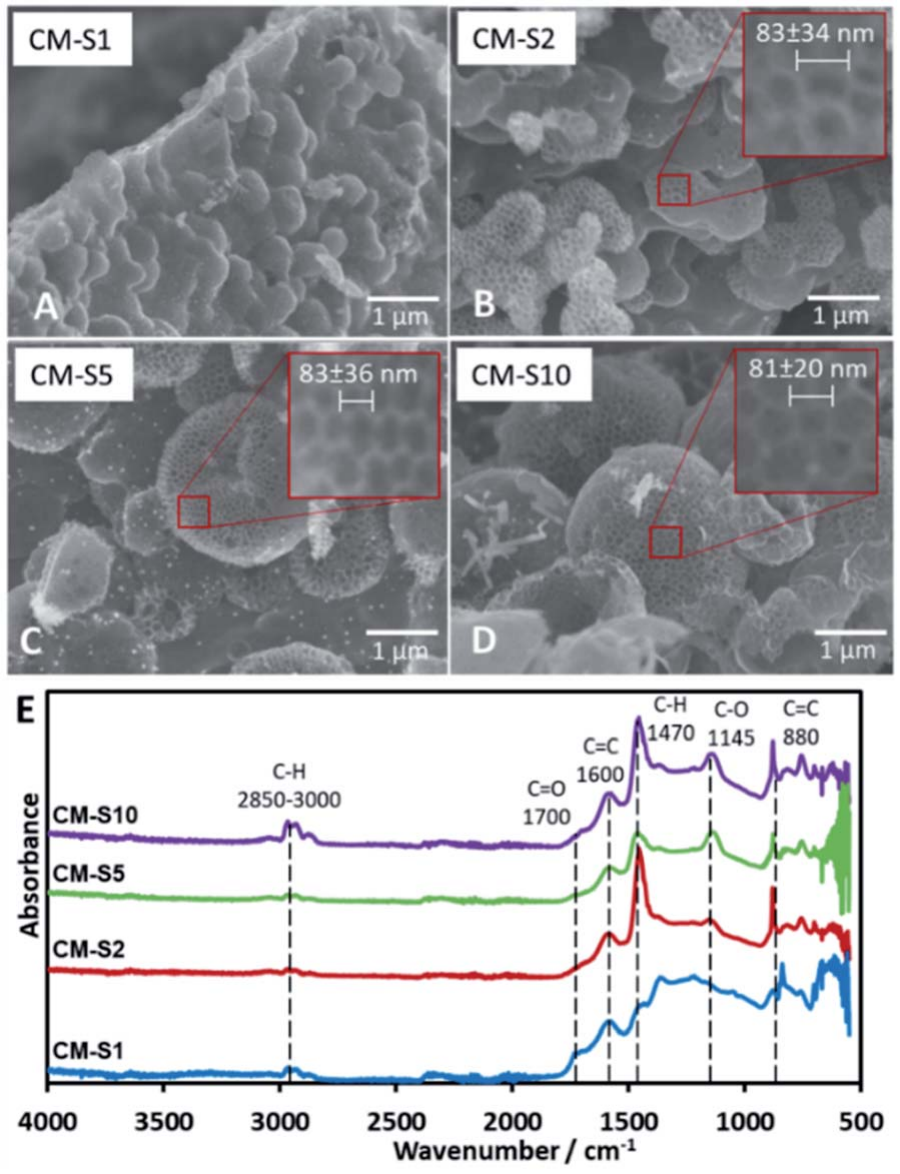
(A–D) SEM images of carbon monoliths with increasing amounts of PSL CM-S1 to CM-S10, (E) IR spectra of CM-S1 to CM-S10.

PSL is the primary template in the system that is removed upon carbonization, forming pores arranged neatly in a honeycomb pattern on the surface of the CM. The maximum amount of PSL that can be loaded into the TOCN matrix before structural failure was investigated. SEM images (Fig. 1) reveal that the minimum amount of PSL required for significant pore formation starts at 32 mg (CM-S2). Pore formation on the surface of CM-S1 is not observed. Too much PSL resulted in a less stable structure as seen in CM-S10 due to the TOCN matrix being spread too thin among the template particles, leading to weaker carbon walls (Fig. 1D). It is also notable that pores with diameter in multiples of the PS particle diameter (*nd*, *n* = 2, 3, …, *d* is the diameter of the PS particle) is not observed, indicating that PSL is stable in the system, does not aggregate, and forms uniform pores on the surface of the material. ImageJ analysis of the pores (Fig. S5†) show a consistent pore size of around 80 nm (Fig. 1A–D), and is slightly larger than the PSL template. Above the lower critical concentration of PSL, the amount of PSL does not affect the final pore size after carbonization. The absence of PSL in the system results to CM without 80 nm pores (Fig. S6†). From the IR spectra (Fig. 1E), the samples are predicted to be composed almost purely of carbon and oxygen, due to the presence of possible C=C (stretching at 1600 cm^−1^, bending at 880 cm^−1^), C=O (stretching at 1700 cm^−1^), and C–O (stretching at 1145 cm^−1^), with little C–H present (stretching at 2850–3000 cm^−1^, bending at 1470 cm^−1^).

### Effect of variation of PF 127 content

**Fig. 2:**
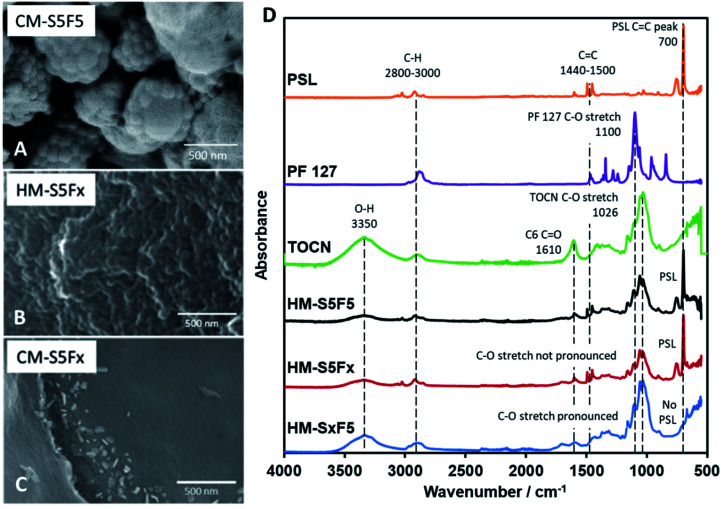
(A) FESEM image of HM-S5F5 showing the inner PF 127 micelle. (B) SEM image of hydrochar without PF 127 added (HM-S5Fx). (C) SEM image of HM-S5Fx after furnace treatment (CM-S5Fx). (D) IR spectra of the polymer components (PSL, PF 127, TOCN), and hydrochars (HM-S5F5, HM-S5Fx, HM-SxF5).

**Fig. 3:**
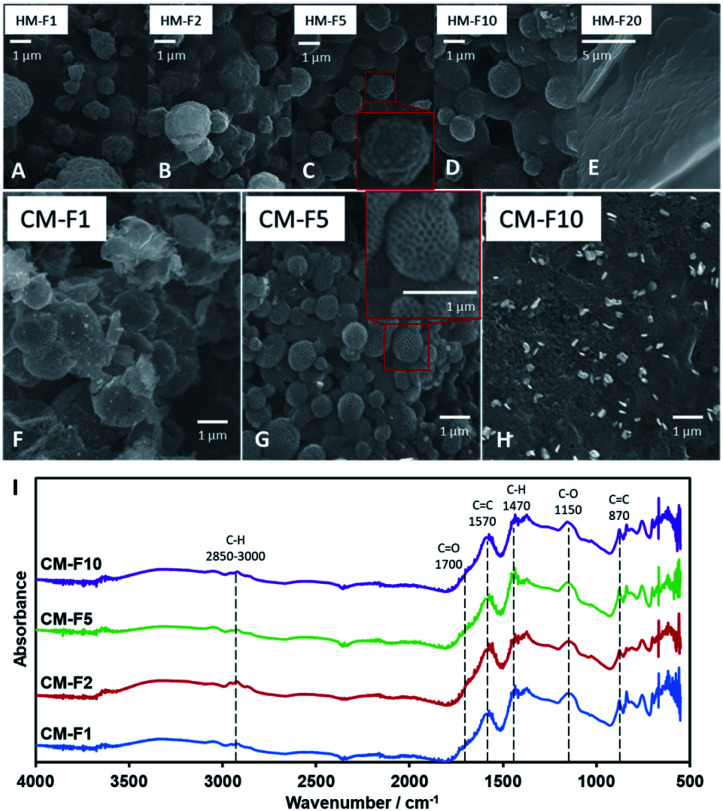
(A–E) SEM images of HM with increasing PF 127 content. (F–H) SEM images of CM with increasing PF 127 content. (I) IR spectra of CM with increasing PF 127 content.

It has been reported by Kubo *et al.* that the concentration of PF 127 has an effect on the macrostructure of the CM by inducing variations in the channels within the material through the thickening of the so-called branches of the bulk material^4^ with glucose. However, using TOCN resulted to a different interaction between TOCN, PF 127, and PSL. PF 127 has an effect during HTT by stabilizing the interface between TOCN and PSL to allow PSL-based templating. PF 127 is also reported to form micelles^35^ that could form the cavities several micrometers across.^4,36^ Other studies using starch^23^ and glucose^37^ have reported distinct carbon spheres,^5,38^ while a study by Zhu *et al.*^39^ reported organic self-assembly of mesoporous carbon spheres from the formation of PF 127 micelles that act as a core for the hydrochar shell during HTT. A similar phenomenon is observed in this study and is confirmed for PF 127 forming micelles 1 μm across (Fig. 2A) that then turn to hollow carbon spheres wherein the carbon shell is dotted with 80 nm pores. To confirm that the micelles are not PSL aggregates, monoliths with 10 to 50 mg PF 127 but with no PSL (CM-SxF1, CM-SxF5 respectively) were synthesized. An increase in the number of 1 μm-sized spheres was observed (Fig. S6†). The templating effect of PF 127 also occurs during HTT, and the structure is preserved after carbonization. In this research, the prevalence of fusion of the carbon spheres to form a monolith, unlike other structures (branched,^4^ distinct spheres^5,38^) is likely due to TOCN fibers entangling and/or one fiber is embedded in adjacent spheres. When PF 127 is not added, HM-S5Fx did not have the 1 μm spheres (Fig. 2B), and after carbonization (Fig. 2C) even the 80 nm pores were absent in CM-S5Fx, showing that the presence of PF 127 as the interface between PSL and TOCN also extends to the pore formation during carbonization. The formation of monoliths even without PSL (CM-S5Fx, Fig. 2C) or PF 127 (CM-SxF5, Fig. S6†) indicates that PF 127 and PSL are not the main nucleating agents in the system. TOCN through fiber entanglement, crosslinking, and hydrothermal degradation aggregates into a monolith during HTT. IR spectra of HM (Fig. 2D) shows that PF 127 and the C6 carboxy group of TOCN are preserved even after HTT with the pronounced C–O stretch at the 1050–1100 cm^−1^ region, and the presence of a C=O peak at the 1700 cm^−1^ respectively. PSL is also intact, just coated with the TOCN nanofibers that turned to hydrochar (Fig. 3A). The amount of PF 127 changes the apparent macroporosity of the material, as well as the density of the unique 1 μm spheres covered in PSL.

The amount of PF 127, shown from samples HM-F1 to HM-F10 affect the density and the uniformity of the spherical shape of the 1 μm spheres, but at 200 mg (HM-F20) too much PF 127 will contribute a significant mass and volume to the hydrochar matrix, covering the structures formed with PSL. After carbonization (Fig. 3B) CM-F1 and CM-F5 exhibited the hollow spheres with a honeycomb surface, while CM-F10 did not, indicating that 100 mg is also too much PF 127, which could be from (1) high gas evolution rate affecting structure stability, and (2) significant amounts of PF 127 leading to carbon contribution to the monolith, altering the structure made with carbon only from TOCN. The IR spectra (Fig. 3I) shows similar peaks as that of samples with increasing PSL content, indicating that PF 127 and PSL both do not significant affect the final functional groups present in the carbon monolith. Peaks for C=C bending at 870 cm^−1^, stretching at 1570 cm^−1^, C=O stretching at 1700 cm^−1^, and ether C–O stretching 1150 cm^−1^ point out highly unsaturated carbon oxidized with oxygen. Little C–H is also observed with stretching at 2850–3000 cm^−1^, and bending at 1470 cm^−1^.

### Effect of hydrothermal treatment duration and temperature

**Fig. 4:**
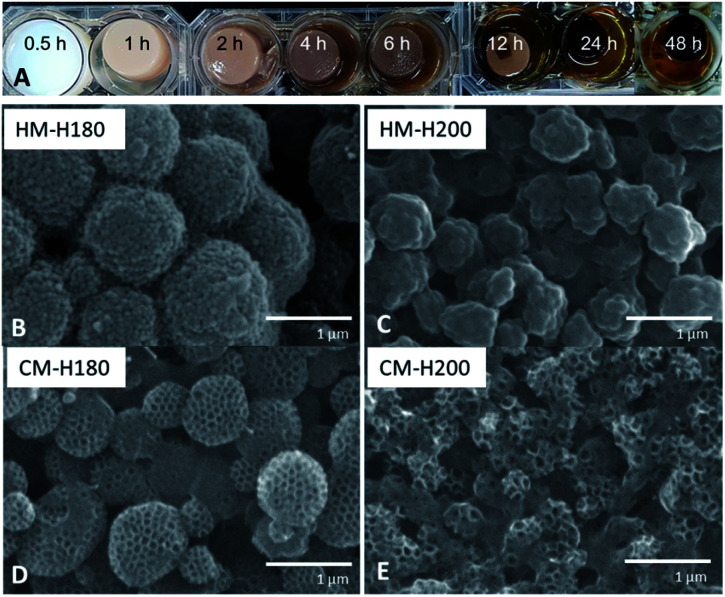
(A) Images of the monolith formed after varying HTT time. (B and C) SEM images of monolith formed with HTT at 180 °C (HM-H180) and 200 °C (HM-H200). (D and E) SEM images of monolith formed at 180 °C (CM-H180) and 200 °C (CM-H200).

From Fig. 4A, it can be observed that HTT when using TOCN has 3 phases: (1) dehydration and heat-induced crosslinking, (2) degradation of polymers into organic compounds such as 5-HMF and phenols and (3) repolymerization, monolith formation, and water release. At 0.5 h, degradation indicated by color change has not yet occurred. A stable monolith was formed by 1 h, indicating that there is sufficient crosslinking *via* esterification or H-bonding to hold the polymer components in place. Phase 2 occurs as degradation of the polymers as the hydrochar turns darker at 2 h, but not all of the organic compounds and PSL have repolymerized back into the hydrochar at 6 h as indicted by the cloudy supernatant. By 12 h, Phase 1 and 2 are complete, and development of Phase 3 continues from 12–48 h when the water becomes clearer, and sufficient water has been removed from the monolith. Because Phase 3 only starts from 12 h, the optimal time will be when Phase 3 has occurred long enough to accumulate majority of the mass from the reagents to get high yield. Only at 48 h did the supernatant become transparent, and thus was considered as the optimal reaction time.

Investigating the effect of increasing HTT temperature revealed that increasing the temperature from 180 °C (HM-H180) to 200 °C (HM-H200) affected the size of the PF 127-based sphere from 1 μm to 500 nm (Fig. 4B and C). It is suggested that polymers that nucleate to form spheres under HTT can be described by the La Mer Nucleation Model.^40,41^ Except for the change in size, the structure remains the same, suggesting the same process occurs at both temperatures. Carbonizing the monoliths at 500 °C formed the same type of structure as well, but cavities between spheres of CM-H200 are smaller than that of CM-H180.

### Effect of carbonization temperature

**Fig. 5:**
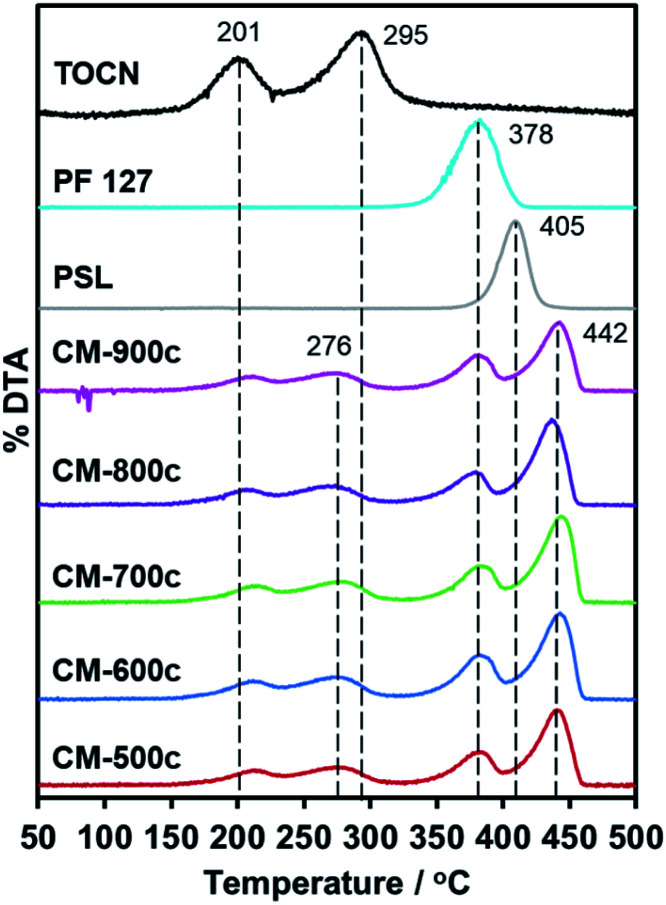
First derivative of the TGA curve (DTA) of CM-500c to CM-900c, PSL, PF 127, and HM-TOCN. No peaks observed above 450 °C, indicating no mass loss.

Replication of furnace conditions in TGA (Fig. 5) show that hydrochar mass loss occurs at two distinct temperatures: 201 °C and 295 °C. The peak at 201 °C corresponds to acid-catalyzed dehydration described as pre-pyrolysis, while 295 °C refers to the complete loss of crystallinity and actual decomposition also called the main pyrolysis.^42^ The main pyrolysis step of hydrochars is 19 °C lower than pure TOCN, indicating lower crystallinity of remaining cellulosic chains. The PF 127 degradation peak at 378 °C does not change with the hydrochar, indicating that PF 127 does not chemically change during HTT. PSL dispersed in the monolith has a higher degradation temperature of 442 °C, unlike pure PSL decomposing at 405 °C. PSL could subsequently react to the carbon matrix formed during the main pyrolysis step of the TOCN component, stabilizing PSL.

**Fig. 6:**
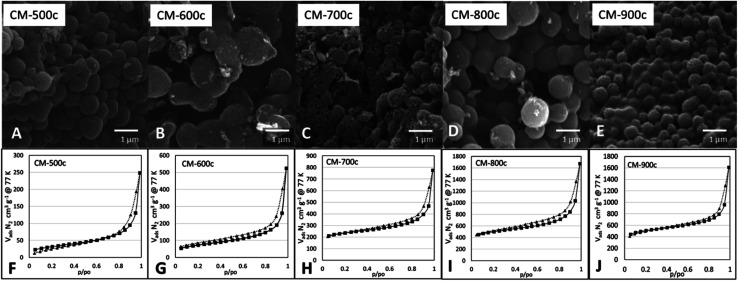
SEM images (A–E) and respective BET N_2_ adsorption and desorption isotherms (F–J) of monoliths carbonized at increasing *T*_hold_ from 500–900 °C.

SEM images at Fig. 6 show a similar structure forming for all monoliths, indicating that the structure formed during HTT is not affected by the carbonization step. The surface area (*S*_BET_) of CM increases significantly as carbonization temperature increases (Table 2) from 109 m^2^ g^−1^ at 500 °C to 1534 m^2^ g^−1^ at 800 °C, then remains relatively the same at 900 °C with 1563 m^2^ g^−1^. The same trend is present for *V*_total_ N_2_ from BJH analysis increasing from 0.396 cm^3^ g^−1^ of CM-500c to 2.1149 cm^3^ g^−1^ of CM-800c, but CM-800c and CM-900c had similar *V*_total_ N_2_ of 2.149 and 2.129 cm^3^ g^−1^ respectively. All CMs show a hybrid of Type IV H1/H3 BET curves, indicating the abundance of relatively uniform mesopores in the material (Fig. 6). The macro- and mesoporosity of CM is formed *via* templating PSL and PF 127, thus the increase in *S*_BET_ is due to the increase in microporosity from increased carbonization temperature as shown in the increasing *V*_micro_ N_2_ from 6.3% of CM-500c to 12.7% of CM-900c. The elemental composition of the monoliths was analyzed *via* XPS, and showed high O/C ratio observed on all CMs. The O/C values are similar to that of methods that use multi-step activation KOH or H_2_O.^6,7^ The decreasing O/C ratio points to oxygen loss due to evolution of gases such as CO and CO_2_. Despite carbon loss from gases however, the carbon yield by mass stays relatively constant from 26.48–30.56%. Carbon yield was calculated by dividing the amount of carbon in the monolith by the amount of carbon in the starting TOCN mass. PSL and PF 127 do not contribute significant amounts of carbon to the material during carbonization, and TOCN is assumed to be sole source of carbon in the system.

**Table 2:** Surface area, product yield, and elemental composition of samples carbonized at different temperatures

	*S*_BET_, m^2^ g^−1^	*V*_total_ N_2_, cm^3^ g^−1^	*V*_meso–macro_ N_2_, cm^3^ g^−1^	*V*_micro_ N_2_, cm^3^ g^−1^	*d*_BJH_ N_2_, nm	Carbon yield mass%	XPS O/C ratio
CM-500c	109.57	0.396	0.371 (93.7%)	0.025 (6.3%)	1.517	28.14 ± 3.63	0.261
CM-600c	250.94	0.802	0.753 (93.9%)	0.049 (6.1%)	1.677	29.65 ± 1.67	0.268
CM-700c	722.55	0.995	0.914 (91.8%)	0.082 (8.2%)	1.432	30.36 ± 3.87	0.222
CM-800c	1534.80	2.149	1.979 (92.1%)	0.170 (7.9%)	1.443	30.56 ± 0.76	0.235
CM-900c	1563.34	2.129	1.859 (87.3%)	0.270 (12.7%)	1.433	26.48 ± 1.81	0.216

**Fig. 7:**
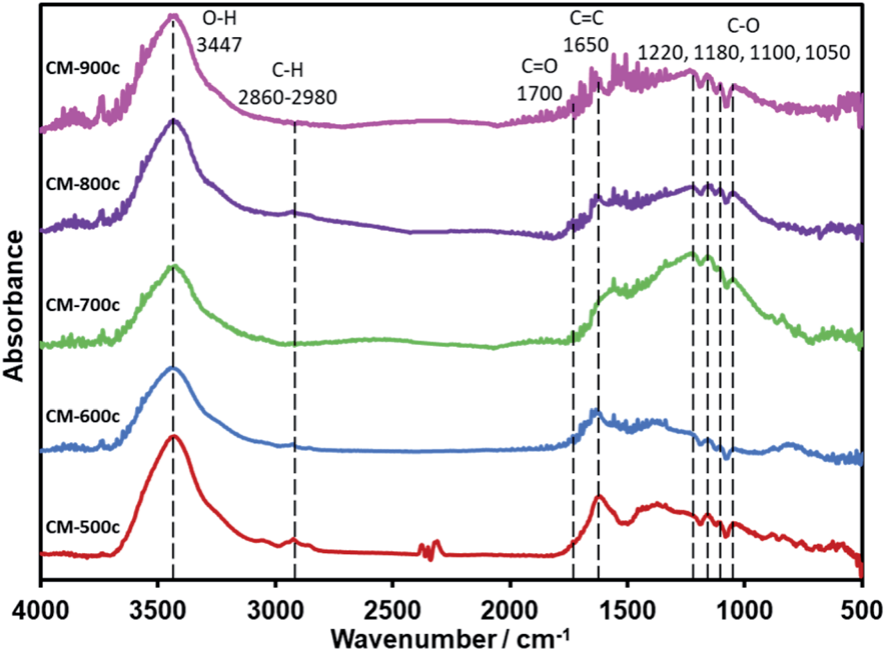
IR spectra of CM-500c to CM-900c powdered and immobilized in KBr pellet.

After the decomposition of the PSL and PF 127 templates, the material undergoes further carbonization until it is made of mostly C and O: C–O at the 1220–1050 cm^−1^ region, C=C at 1650 cm^−1^, and C=O at 1700 cm^−1^ (Fig. 7). While the O–H peak at 3447 cm^−1^ could also be another indicator for O–H groups in the material, differences in mass when the CMs were dried for BET point to adsorption of water vapor to some extent. There is almost no C–H, which is the same as that of CM-S5 (Fig. 1) and CM-F5 (Fig. 3). XPS analysis further confirms the elemental composition showing only peaks of C, O, and indium (XPS stage) (Fig. S7†). Focusing on the C 1s peak shows no clear trend between carbonization temperature and concentrations of the amount of carbon nor the specific oxygen functional groups. The peak at 285.0 eV is known to be graphitic carbon,^43^ and the amount of graphitic carbon steadily decreases from CM-500c to CM-800c from the inclusion of oxygen in the monolith, then increases again at CM-900c which likely due to the higher loss of oxygen from evolution of gases (Fig. S8,†Table 3). C–O in the monolith is resolved in IR as well as XPS. From Fig. 7 ester C–O stretching 1220 cm^−1^, tertiary, secondary, and primary alcohols at 1180, 1100, and 1050 cm^−1^ respectively can be observed. Aliphatic ether C–O peaks are likely merged with the tertiary and secondary alcohol peaks in this region and could not be further resolved. For XPS the relative amount of C–O is shown to be increasing from 15.36% for CM-500c then stabilizing at around 20% for samples CM-600c, CM-700c, and CM-900c. CM-800c had an unnaturally high C–O percent which is likely C–O and C=O being unresolved (Fig. S8†). CM-900c had an additional peak at 293.0 eV which is most likely a π–π* interaction similar to that observed at 950 °C from Yu *et al.*^22^ It should be noted that for Fig. 7, the IR spectra was taken using the KBr pellet method instead of ATR due to low peak intensity observed for CM-700c, CM-800c, and CM-900c. CM-500c and CM-600c could be analyzed using ATR, but CM-500c and CM-600c were analyzed using the KBr pellet method as well for data consistency.

**Table 3:** XPS data of samples CM-500c to CM-900c

	C 1s				O 1s			
	C %	Group	B.E eV	Conc. %	O %	Group	B.E eV	Conc. %
CM-500c	79.29	Graphitic C	285.0	71.53	20.71	C=O	531.5	64.10
		C–O	287.0	15.36		O–C=O	533.2	35.90
		C=O	288.9	4.55				
		O–C=O	290.9	8.56				
CM-600c	78.85	Graphitic C	284.9	61.55	21.15	C–O	532.2	100.00
		C–O	286.7	20.43				
		C=O	288.2	6.92				
		O–C=O	290.5	11.10				
CM-700c	81.86	Graphitic C	285.0	64.22	18.14	C–O	532.0	100.00
		C–O	286.8	19.15				
		C=O	288.4	6.45				
		O–C=O	290.6	10.18				
CM-800c	80.98	Graphitic C	284.9	45.28	19.02	C–O	532.2	80.96
		C–O	286.1	45.09		Adsorbed H_2_O	534.6	10.61
		O–C=O	290.3	9.63		C=O	530.2	8.43
CM-900c	82.25	Graphitic C	284.5	59.52	17.75	C–O	532.0	94.35
		C–O	286.5	19.72		C=O	529.7	5.65
		C=O	288.1	9.02				
		O–C=O	290.5	8.39				
		Plasmon loss	293.0	3.34				

### Oil–water separation adsorption capacity

The synthesized honeycomb-like pore geometry is useful in increasing the packing density of mesopores and macropores of the carbon surface, increasing multi-modality. This hierarchical pore structure helps in water penetration, increasing contact in the bulk of the monolith, and subsequently enhancing performance for batch adsorption applications. A fossil fuel standard of octanoic acid and isooctane based on an oil and grease content in water determination method^34^ was used to evaluate the adsorption capacity (*q*_e_) of the monolith. *q*_e_ of 9.6 to 11.3 g g^−1^ were observed, with CM-500c having the highest adsorption capacity (Table 4). The theoretical maximum adsorption capacity is 12.922 g g^−1^ (total amount of oil in oil–water mixture divided by 0.02 g carbon), thus CM-500c has an experimental capacity efficiency of 90.31%. The effect of carbonization temperature *vs.* adsorption capacity has a peculiar trend where *q*_e_ decreases as the carbonization temperature increases from 500 to 700 °C, but *q*_e_ increases from 700 to 900 °C.

**Table 4:** Carbonization temperature *vs.* adsorption performance at 5 mg mL^−1^

Temp, 5 mg mL^−1^	Ave *q*_e_, g g^−1^	Water contact angle
500 °C	11.3 ± 0.9	98.3° ± 1.4
600 °C	9.9 ± 1.7	113.0° ± 5.5
700 °C	9.6 ± 0.8	121.5° ± 4.0
800 °C	10.3 ± 0.2	103.7° ± 4.7
900 °C	10.7 ± 0.1	101.7° ± 2.2

For porous materials the mechanism for organic phase adsorption changes whether it is the exterior or the interior of the bulk material. Electrostatic interaction in the interface of the carbon, and water allowing for the adsorption of micellar particles occurs at the exterior surface of the monolith. There is a clear decrease in the O/C ratio between 600 °C and 700 °C. The hydrophobicity of the material increases with increasing carbonization temperature, which explains that even with low surface area, CM-500c has a very high *q*_e_ resulting from lower surface tension between the monolith and water. Water contact angle can be used as a rough indicator of hydrophobicity for bulk materials,^44^ and the inverse trend of increasing then decreasing hydrophobicity is observed (Table 4). The rugged surface of the monolith further increases the surface area, increasing exterior adsorptive capacity. The interior however is usually inaccessible due to trapped air, and in general the smaller the pores, the more time it takes for water to replace air in the monolith due to high surface tension,^45^ leading to a decrease in the effective adsorptive surface of the monolith. The hierarchical porous nature of the monolith, namely the macropores, however makes the monolith take in water relatively easier, as observed with the mass of the monolith after oil adsorption (ave. 0.59 g) far heavier than the dry monolith (ave. 0.02 g) and the theoretical maximum of oil (0.26 g) combined. Given that the macropores are due to the templating from the PSL, the degree of macroporosity for all the monoliths are relatively similar. It is also known that the observed increase in surface area is due to increased microporosity formed during higher carbonization temperature, increasing the adsorption capacity as observed for CM-900c. A similar method of inducing small spaces within the carbon structure increasing adsorption capacity has been reported but in using expanded graphite to increase adsorption capacity.^46^ Even though a less significant factor compared to hydrophobicity, higher surface area allows CM-900c to have a high *q*_e_.

1 Linear pseudo-first order ln(*q*_e_ − *q*_*t*_) = ln *q*_e_ − *k*_PFO_*t*

2
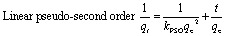


**Fig. 8:**
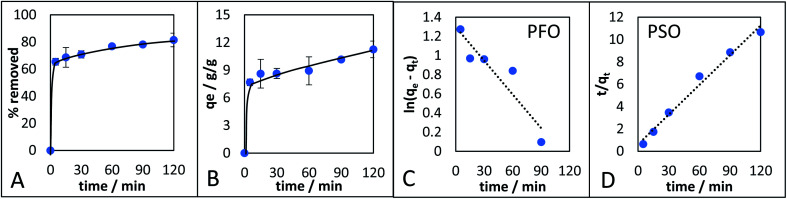
CM-500c adsorption kinetic studies. (A) Percent removal of octanoic acid and isooctane with increasing time, (B) *q*_e_ over time, (C) pseudo-first order model (PFO), and (D) pseudo-second order model.

The adsorption kinetics of the monolith with the highest *q*_e_ (CM-500c) was investigated (Fig. 8). CM-500c shows rapid adsorption reaching 65.4% removal in 5 min, and maximum removal reached was 81.15% with *q*_e_ of 11.3 after 2 h (Fig. 8A). The *q*_e_ changed by 1.4% after 24 h 11.4g g^−1^, indicating that adsorption equilibrium as already achieved by 2 h. Pseudo-first order (PFO) and pseudo-second order (PSO)^47,48^ are the empirical kinetic models that give the rate of the adsorption. For PFO (eqn (1)), the plot of ln(*q*_e_ − *q*_*t*_) *vs. t* (Fig. 8C) where *q*_e_ is the adsorption capacity in equilibrium (g g^−1^), *q*_*t*_ is the adsorption capacity at a given time (g g^−1^), *k*_PFO_ is the rate of adsorption calculated from PFO (g g^−1^ min^−1^), and *t* is time in min, shows poor correlation, and likewise a different calculated adsorption capacity at equilibrium (*q*_e, cal_) of 3.6450 compared to the theoretical adsorption capacity at equilibrium (*q*_e, exp_) at 11.2554. Using eqn (2) as the linearized form of the PSO equation on the other hand has very good correlation of *R*^2^ = 0.9842 and comparable *q*_e, exp_ = 11.2986 g g^−1^ (Table 5, Fig. 8D). The rate of adsorption from PSO (*k*_PSO_) is similar to *k*_PFO_, showing agreement with the general adsorption rate performance of CM-500c. Possible physical interpretations of PFO and PSO were elucidated,^48–50^ and two of the conditions where a general kinetic model becomes PFO or PSO are based on (1) the active sites of the adsorbent as the rate-limiting step – *i.e.* a function of the affinity of the adsorbate to the adsorbent, and the (2) the number of active sites of the adsorbent. Hydrophobic adsorbates with hydrophobic adsorbents are better represented by PSO, reflecting a high number of active sites or sites of adsorption in the monolith. Further studies on the adsorption kinetics of the material are needed to elucidate effect of surface functionalization on adsorption.

**Table 5:** Adsorption kinetic parameters from CM-500 at 298 K

	*q*_e, exp_ (g g^−1^)	*k* (g g^−1^ min^−1^)	*q*_e, calc_ (g g^−1^)	*R* ^2^
Pseudo-first order (PFO)	11.2554	0.0117	3.6594	0.8622
Pseudo-second order (PSO)	11.2554	0.0124	11.2986	0.9842

The adsorption capacity of CM-500c is compared to other carbon structures, studies with similar microsphere structure, and biomass sources (Table 6). Carbon nanotubes (CNTs),^51^ multi-walled carbon nanotubes (MWCNTs),^52^ graphene oxide (GO),^53^ and graphite^54^ and showed much higher adsorption capabilities, but major disadvantages are slower or more expensive methods such as chemical vapor deposition (CVD), and use non-renewable materials. A study has reported a similar structure with carbon microspheres with rough outer surfaces,^55^ but also utilize CVD. A study with similar carbonization conditions using cellulosic carbon precursor^33^ but without template-controlled porosity reported higher surface area, but lower *q*_e_. In general, biomass-based sorbents fare poorer than advanced carbon materials.^56^ If PSL and PF-127 can be stabilized in the natural carbon precursors, then it can be a promising way of increasing the performance of cheap and renewable carbon from biomass.

**Table 6:** Other reported carbon materials and their respective adsorption capacity

Adsorbent	Carbon synthesis method	Adsorbate	Surface area (m^2^ g^−1^)	Adsorption capacity *q*_e_ (g g^−1^)	Reference
PSL-templated carbon monolith from cellulose nanofiber (this study)	Hydrothermal carbonization	Isooctane and octanoic acid in water	109.57	9.6–11.3	
Activated carbon pellets	—	Isooctane and octanoic acid in water	912.83	6.3–8.8	
CNT sponges	CVD	Oils and solvents	300–400	80–180	Gui *et al.*^51^
MWCNT-coated PU foam	—	Oils and solvents	—	20–60	He *et al.*^52^
GO-coated PAN foam	—	Oils and solvents	900–1100	80–201	Feng *et al.*^53^
Exfoliated graphite	Acid treatment	Heavy oil	158	9.7 L/100 g	Hristea *et al.*^54^
Raw graphite	—		1.6	0.1 L/100 g	
Carbon microspheres	CVD	Oleic acid in water	—	19	Bakhshi *et al.*^55^
Magnetic porous carbon aerogel from popcorn	Fe(NO_3_)_3_·9H_2_O with carbonization	Corn oil	229.25	10.28	Dai *et al.*^33^

Lastly CM-500c was compared to commercially available activated carbon pellets using the same adsorption experiment method. The O/C ratio is comparable at 0.247 with 5.36% difference from CM-500c (Table S1†). *S*_BET_ is much higher at 912.83 m^2^ g^−1^ which is due to the high amount of micropores, comprising 72.8% of *V*_total_ N_2_. High microporosity could also be seen in the BET isotherm (Fig. S9†), where the sharp increase in *V*_ads_ N_2_ at *p*/*p*_o_ = 0–0.3 as well as the Type IV H4 hysteresis loop point to non-uniform micropores in the material. Despite the high *S*_BET_ and the abundance of micropores, the commercially available activated carbon pellets fared worse than the monolith at 8.8 g g^−1^ (60.69% removed), indicating that the unique porous structure formed from PS templating is highly influential to the adsorption of adsorbates into the bulk of the carbon monolith, thereby resulting to an increase in performance.

On the scalability of the synthesis method, the size of the hydrothermal vessel will have an effect on the integrity of the carbon monolith. A larger vessel could allow larger hydrochars to be made without changes in the structure and homogeneity of the system because even though TEMPO-oxidized cellulose nanofiber (TOCN) dispersions are viscous even at 1%, mixing for 1 h ensures homogeneity. Pluronic F-127 and the polystyrene latex (PSL) are easier to disperse in aqueous systems, thus posing no problems either. The observed optimal hydrothermal treatment time is 48 h, ensuring that sufficient time is given to reach 180 °C and not pose problems in uneven heat distribution in the system, and in hydrochars undergoing the proposed phases of hydrothermal degradation and subsequent aggregation. With regards to the carbonization step, it is shown in the dTG curves of the monoliths (Fig. 5) that degradation of the polymer components also occurs in distinct phases, with no significant overlap in their respective degradation temperatures. This prevents over-expulsion of gases that might pose a risk to the monolith breaking apart. Due to these reasons, there would be little risk of a larger monolith collapsing due to changes in size from multiple sections of the material either carbonizing or thermally degraded.

## Conclusions

A carbon monolith comprised of fused hollow spheres with an open skeletal pore structure resembling a honeycomb pattern was synthesized using TEMPO-oxidized cellulose nanofibers as the carbon source. The structure formed only during HTT, which does not change during carbonization. The increase in surface area is from the increased microporosity from the high carbonization temperature, notably from 700–900 °C. While PSL is responsible for the formation of the honeycomb pattern on the surface of the spheres, the integrity of the structure as well as the template for the larger fused spheres is due to the presence of PF 127 acting as an interface, and as a precipitate micelle embedded in the TOCN matrix respectively. The hierarchical and open porous network observed is deemed good for adsorption of hydrophobic liquids due to the high carbon content and low XPS O/C ratio, thus used for oil–water separation. Using carbonization temperature-induced changes in surface area and hydrophobicity, CM-500c to CM-900c were tested for oil adsorption and was found that CM-500c had the highest adsorption capacity of 11.3 g g^−1^, next to CM-900c of 10.7 g g^−1^. Combining the multiple methods of optimizing structure and hydrophobicity through hydrothermal carbonization as well as templating from PSL and PF 127 resulted in a very high adsorption capacity of the carbon monolith.

## Conflicts of interest

There are no conflicts to declare.

## Supplementary Material

RA-011-D0RA08950H-s001
