# Supplementary material for: Fused sphere carbon monoliths with honeycomb-like porosity from cellulose nanofibers for oil and water separation
Source: RSC Adv. 2021 Jan 8;11(4):2202–12. doi: 10.1039/d0ra08950h (PMC8693729; doi:10.1039/d0ra08950h)
Supplement: RA-011-D0RA08950H-s001 [file RA-011-D0RA08950H-s001.pdf]

Electronic Supplementary Material (ESI) for RSC Advances.
This journal is © The Royal Society of Chemistry 2020

Supplementary Information

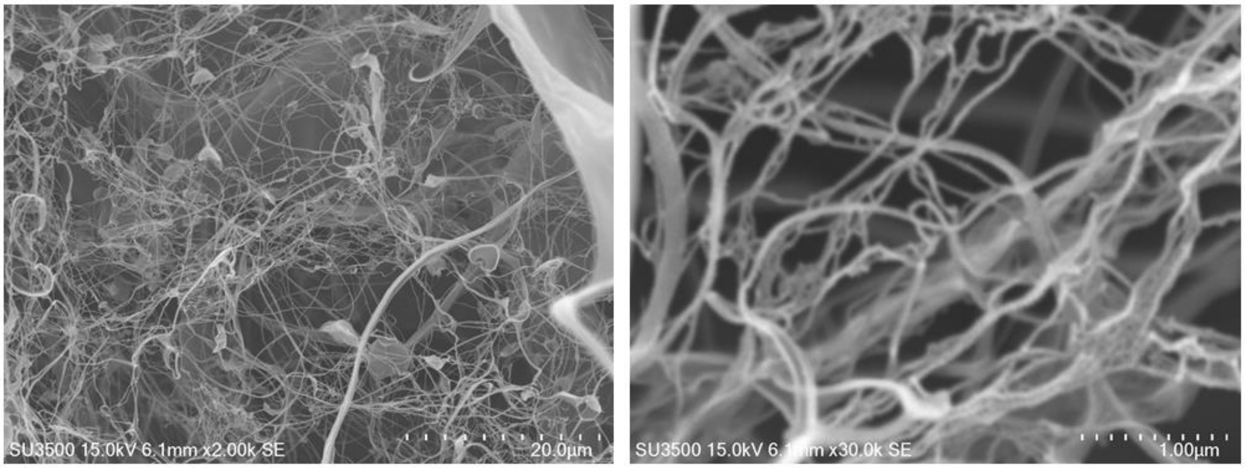

Fig. S 1. SEM images of TOCN.

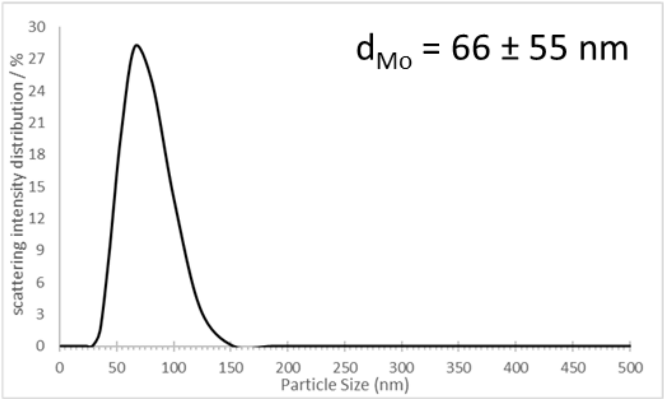

Fig. S 2. DLS curve of PSL.

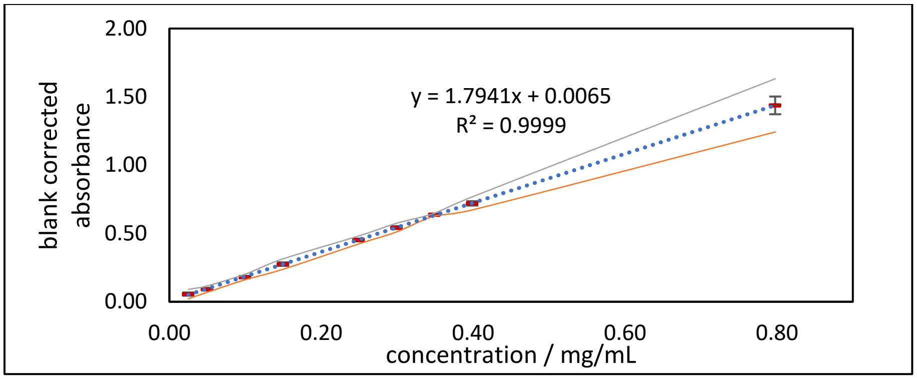

Fig. S 3. Calibration curve. The calibration curve passes a 2-tailed t-test with $\alpha = 0.05$ and a $p = 3.96 \times 10^{-16}$, well below 0.05, proving that the calibration curve is statistically sound and reproducible. The calibration curve standard solutions are: 0.025, 0.050, 0.100, 0.150, 0.250, 0.300, 0.350, 0.400, and 0.800 mg/mL.

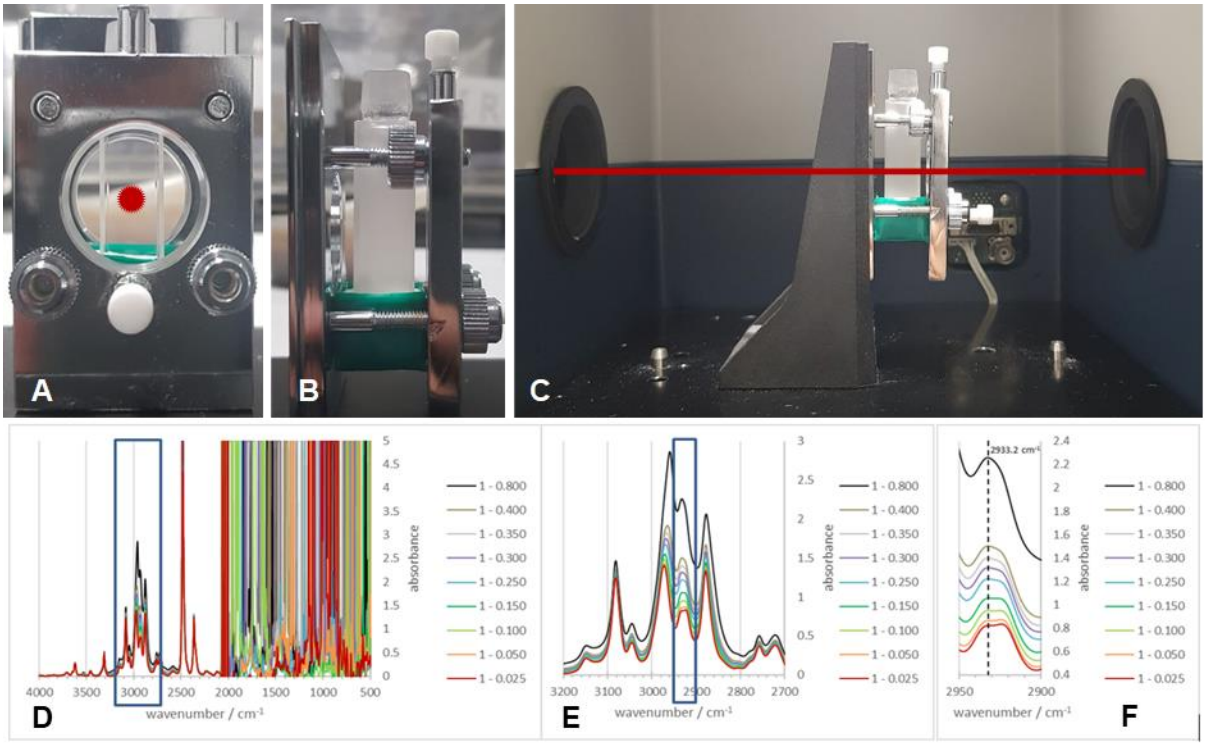

Fig. S 4. Liquid IR setup. Images of the setup of the glass cell in the FTIR chamber: A) front view, B) side view, C) entire chamber setup. The green handle at the bottom of the cuvette is made of foam to prevent contact of the cuvette with the metal, avoiding breakage of the cuvette. The IR beam is at the center of the circular cutout, and the foam holder does not interfere with the beam (red). IR spectra of the calibration curve standards: D) 4000-400 cm^{-1}, E) 3200-2700 cm^{-1}, F) 2950-2900 cm^{-1}.

Infrared spectroscopy was used to make the calibration curve, following strict analytical cleaning and decontamination between each spectrum recording. 3 mL of the calibration standard was placed in a quartz cell with a path length of 10 mm, then analyzed with the following FTIR conditions: 8 cm^{-1} resolution, 64 scans, absorbance mode, and range from 4000-400 cm^{-1}. The quartz cell is IR-opaque from 2200-400 cm^{-1} and unstable from 2500-2200 cm^{-1}, but was still reported to compare and confirm that the quartz cell has a stable, IR-transparent region. The region used for analysis was the 3200-2700 cm^{-1} region, and the peak used for curve fitting is the 2933.2 cm^{-1} peak.

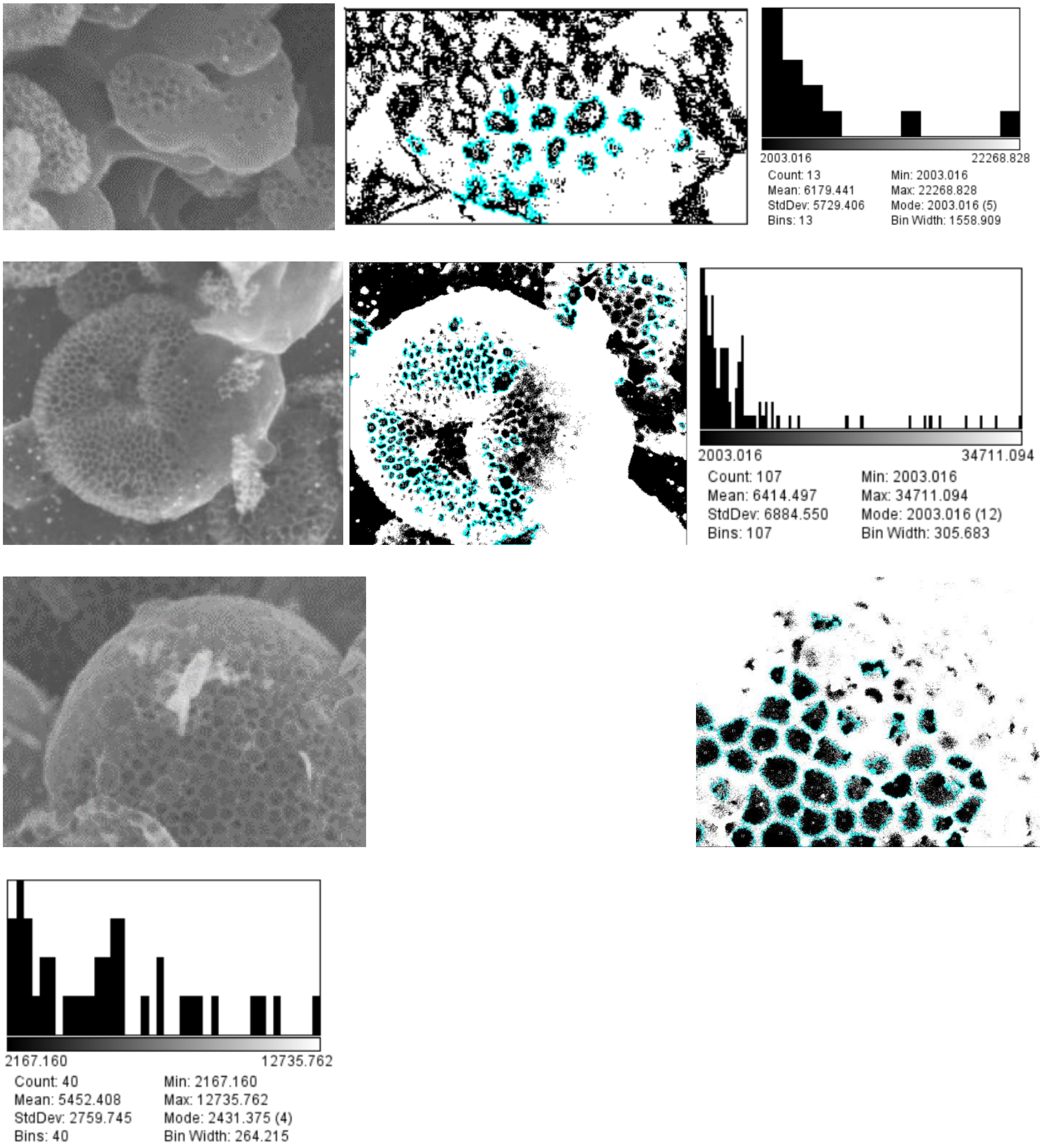

Fig. S 5. ImageJ analysis: original SEM image (left), overlay mask (middle), and histogram (right) of A) CM-S2, B) CM-S5, and C) CM-S10.

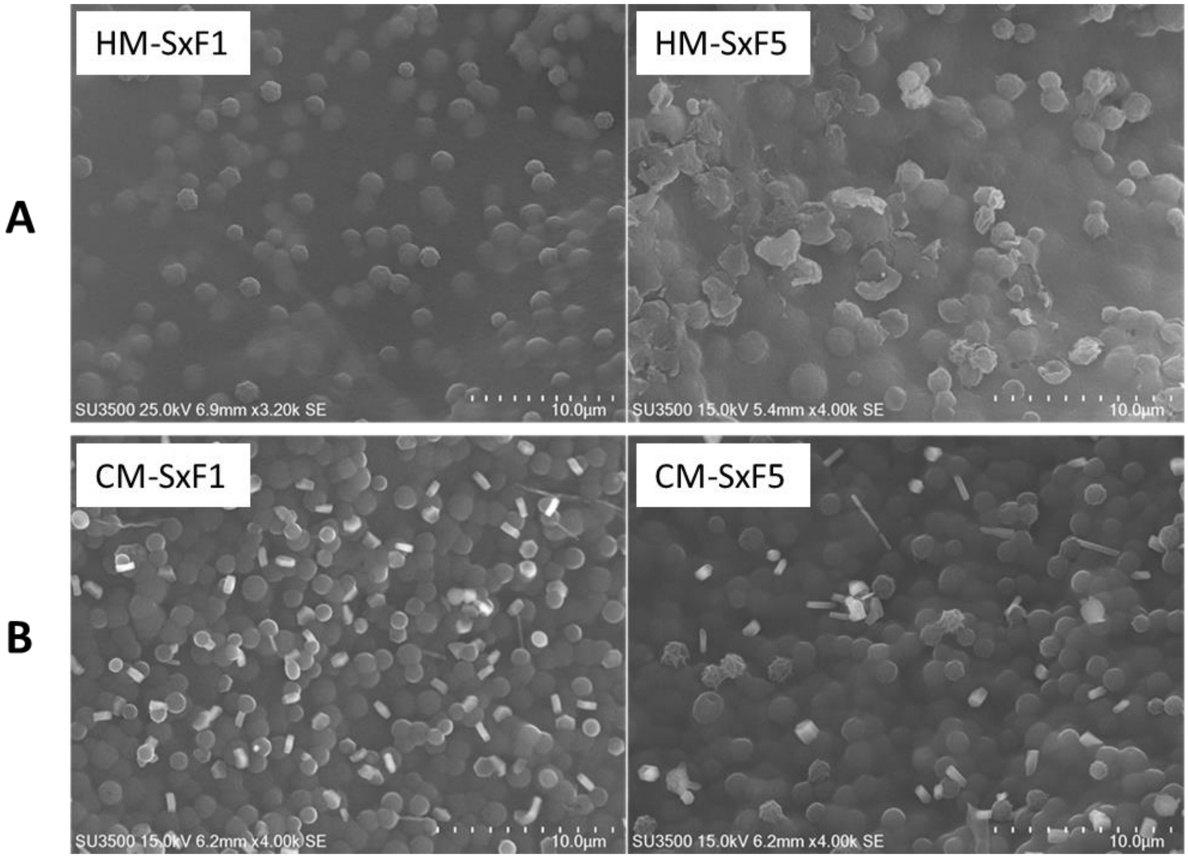

Fig. S 6. SEM images of monoliths with no PSL and varied PF 127 content: A) hydrochar of HM-SxF1 and HM-SxF5, and B) carbon monolith of CM-Sx-F1 and CM-SxF5.

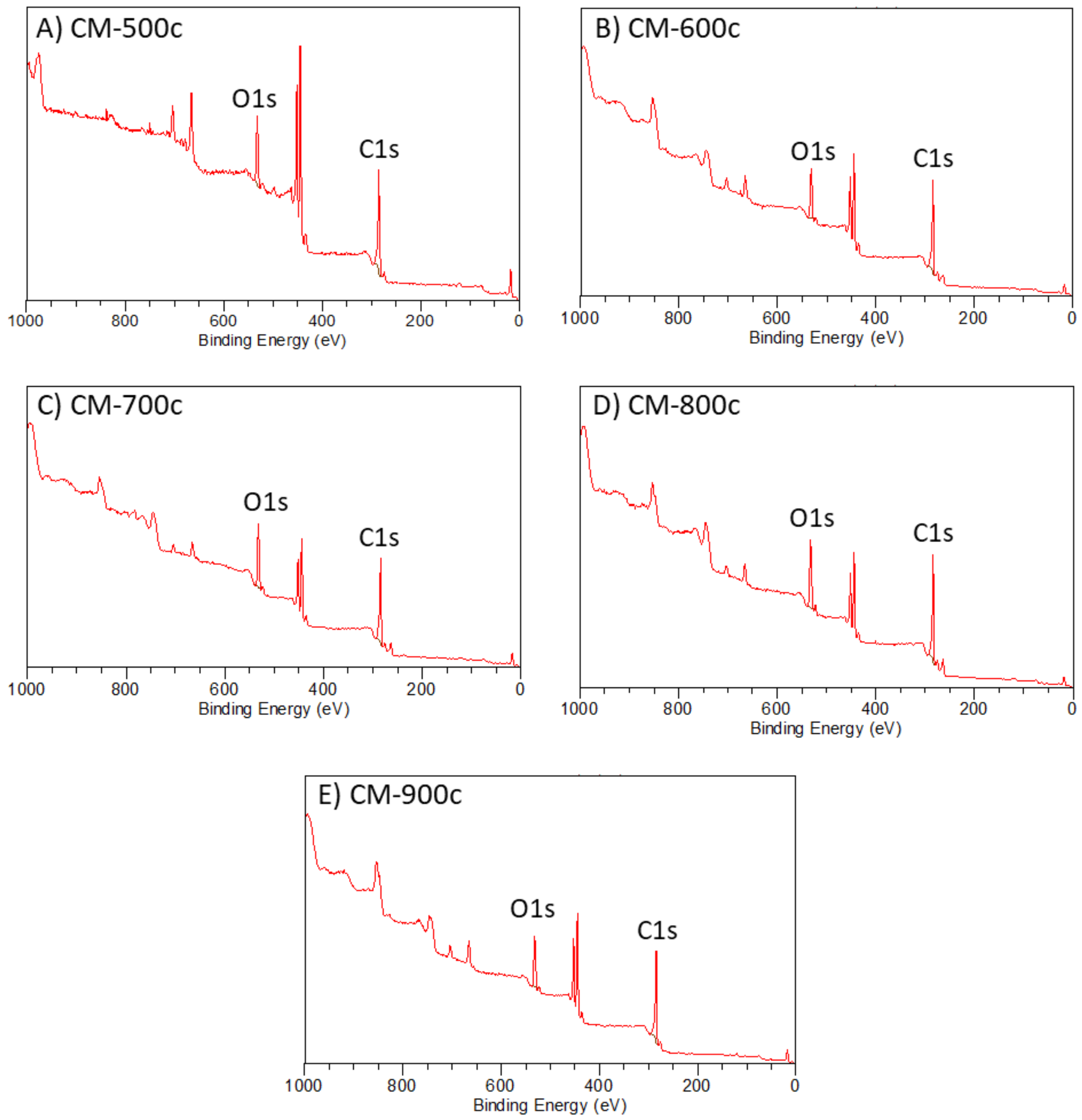

Fig. S 7. XPS spectra of CM-500c to CM-900c.

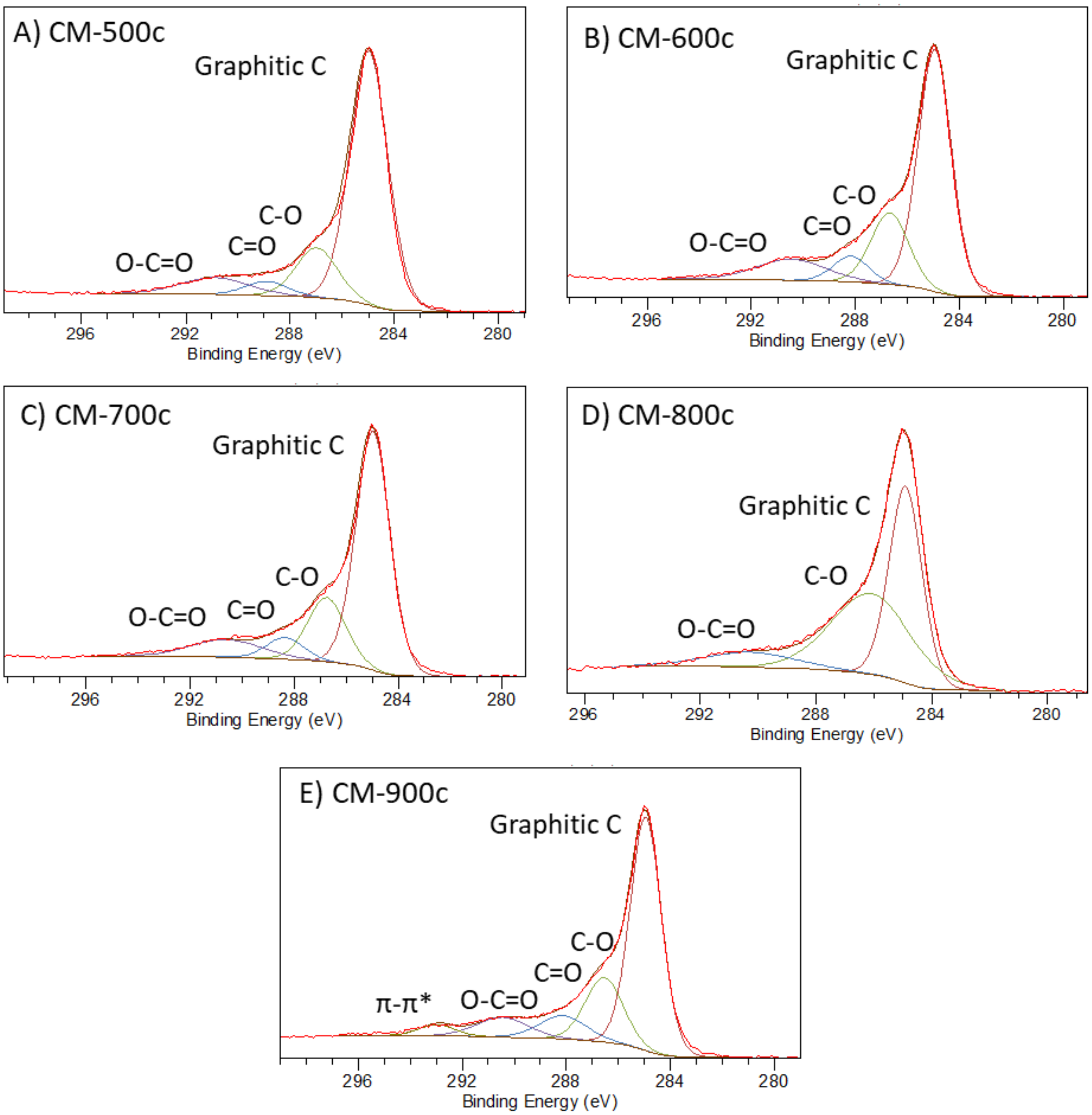

Fig. S 8. C1s XPS spectra of CM-500c to CM-900c

Table S 1. Surface area and adsorption capacity characterization of commercially available carbon pellets.

S_{BET} $m^2\ g^{-1}$	V_{total} N_2 $cm^3\ g^{-1}$	$V_{meso\text{-}macro}$ N_2 $cm^3\ g^{-1}$	V_{micro} N_2 $cm^3\ g^{-1}$	d_{BJH} N_2 nm	O/C ratio	$q_{e,\ exp}$ $g\ g^{-1}$	$q_{e,\ calc}$ $g\ g^{-1}$	k $g\ g^{-1}\ min^{-1}$	PSO R^2
912.83	0.139	0.038 (27.2%)	0.101 (72.8%)	1.461	0.247	8.7601	8.4413	0.0188	0.9666

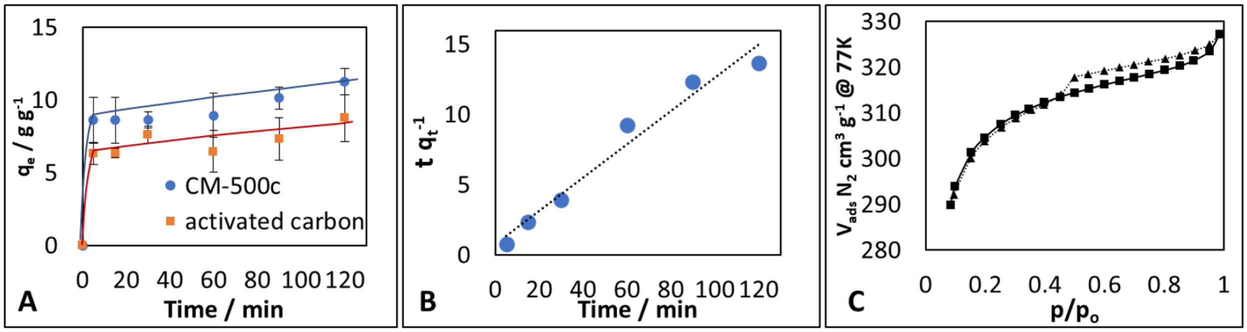

Fig. S 9. A) Comparison of activated carbon and CM-500c in adsorption capacity q_e vs time, B) Pseudo-second order (PSO) model fitting of q_e of the activated carbon with R^2 = 0.9666, and C) BET isotherm of the activated carbon.
